# Redefining the Clostridioides difficile σ^B^ Regulon: σ^B^ Activates Genes Involved in Detoxifying Radicals That Can Result from the Exposure to Antimicrobials and Hydrogen Peroxide

**DOI:** 10.1128/mSphere.00728-20

**Published:** 2020-09-16

**Authors:** Ilse M. Boekhoud, Annika-Marisa Michel, Jeroen Corver, Dieter Jahn, Wiep Klaas Smits

**Affiliations:** a Department of Medical Microbiology, Leiden University Medical Center, Leiden, The Netherlands; b Centre for Microbial Cell Biology, Leiden, The Netherlands; c Netherlands Centre for One Health, Leiden, The Netherlands; d Braunschweig Integrated Centre of Systems Biology (BRICS), Technische Universität Braunschweig, Braunschweig, Germany; e Institute of Microbiology, Technische Universität Braunschweig, Braunschweig, Germany; University of Iowa

**Keywords:** *Clostridium difficile*, antimicrobial agents, *in vitro* transcription, luciferase, regulon, sigma factors

## Abstract

In many Gram-positive bacteria, the general stress response is regulated at the transcriptional level by the alternative sigma factor sigma B (σ^B^). In C. difficile, σ^B^ has been implicated in protection against stressors such as reactive oxygen species (ROS) and antimicrobial compounds. Here, we used an anti-σ^B^ antibody to demonstrate time-limited overproduction of σ^B^ in C. difficile despite its toxicity at higher cellular concentrations. This toxicity eventually led to the loss of the plasmid used for anhydrotetracycline-induced σ^B^ gene expression. Inducible σ^B^ overproduction uncouples σ^B^ expression from its native regulatory network and allows for the refinement of the previously proposed σ^B^ regulon. At least 32% of the regulon was found to consist of genes involved in the response to reactive radicals. Direct gene activation by C. difficile σ^B^ was demonstrated through *in vitro* runoff transcription of specific target genes (*cd0350*, *cd3614*, *cd3605*, and *cd2963*). Finally, we demonstrated that different antimicrobials and hydrogen peroxide induce these genes in a manner dependent on this sigma factor, using a plate-based luciferase reporter assay. Together, our work suggests that lethal exposure to antimicrobials may result in the formation of toxic radicals that lead to σ^B^-dependent gene activation.

**IMPORTANCE** Sigma B is the alternative sigma factor governing stress response in many Gram-positive bacteria. In C. difficile, a *sigB* mutant shows pleiotropic transcriptional effects. Here, we determine genes that are likely direct targets of σ^B^ by evaluating the transcriptional effects of σ^B^ overproduction, provide biochemical evidence of direct transcriptional activation by σ^B^, and show that σ^B^-dependent genes can be activated by antimicrobials. Together, our data suggest that σ^B^ is a key player in dealing with toxic radicals.

## Abstract (precis)

Sigma B is the alternative sigma factor governing stress response in many Gram-positive bacteria. In C. difficile, a *sigB* mutant shows pleiotropic transcriptional effects. Here, we determine genes that are likely direct targets of σ^B^ by evaluating the transcriptional effects of σ^B^ overproduction, provide biochemical evidence of direct transcriptional activation by σ^B^, and show that σ^B^-dependent genes can be activated by antimicrobials. Together, our data suggest that σ^B^ is a key player in dealing with toxic radicals.

## INTRODUCTION

Disruption of the normal gastrointestinal flora as a result of antimicrobial treatment can lead to a *Clostridioides* (Clostridium) *difficile* infection (CDI) (1). Clostridioides difficile is a Gram-positive, spore-forming obligate anaerobe and the primary cause for nosocomial infectious diarrhea (2). Its highly resistant endospores are usually transmitted via the oral-fecal route and germinate into vegetative cells in the colon upon contact with primary bile acids and other inducing factors (3). In the gut, vegetative C. difficile cells face many environmental stressors, including variations in oxygen tension, pH, osmolarity, nutrient availability, and the inflammatory responses of the immune system (4). The bacteria are also faced with antimicrobial compounds that are produced by the host, the resident microbiota, or given externally during medical therapy (5). The physiological response of C. difficile to these insults and the inflammatory responses triggered by CDI can result in the production of reactive oxygen species (ROS), reactive nitrogen species (RNS), and nitric oxide (NO) (2, 6).

Bacteria need to adapt to changing environmental conditions, including stresses, by adapting their physiology in a timely manner. This is achieved by fast transcriptional reprogramming, followed by briefly delayed changes at the translational level (7). The alternative sigma factor sigma B (σ^B^, encoded by the *sigB* gene), which regulates the general stress responses in a variety of Gram-positive organisms, is central to the maintenance of cellular homeostasis during stress adaptation (8, 9).

Sigma factor B activity in Firmicutes species is regulated at the protein level by a partner-switching mechanism in which the anti-sigma factor RsbW binds and inhibits σ^B^ association with the RNA polymerase under nonstressed conditions. When a σ^B^-activating stress is sensed, the dephosphorylated anti-anti-sigma factor RsbV sequesters RsbW, allowing for the association of free σ^B^ with the RNA polymerase core enzyme (8, 10). In C. difficile, the phosphatase RsbZ is responsible for RsbV dephosphorylation (11). The tight regulation of σ^B^ activity by a partner-switching mechanism is necessary, as the energy burden associated with σ^B^ activity was found to be disadvantageous in several different organisms (12, 13).

Despite the burden associated with its expression, σ^B^ is essential for survival for several pathogenic bacterial species in response to host-dependent stressors or antimicrobials. For example, in Listeria monocytogenes, σ^B^ is involved in counteracting the effects of the acidic pH encountered in the stomach and upon invasion of intestinal epithelial cells in the lysosome (14, 15). In Staphylococcus aureus, σ^B^ overproduction leads to thickening of the cell wall and increased resistance to beta-lactam antimicrobials (16). The *sigB* homologue *sigF* of Mycobacterium tuberculosis is induced by small amounts of rifamycin (17). Analogously, Bacillus subtilis σ^B^ is involved in resolving a rifampin-induced growth arrest (18). There is also evidence for the involvement of σ^B^ in C. difficile in the response to antimicrobial substances. Mutants of *sigB* show increased susceptibility to rifampin and mitomycin C and are also more sensitive to hydrogen peroxide, nitroprusside, and di‐ethylamine NONOate (19). However, the underlying molecular mechanisms remain unknown. Finally, indirect activation of σ^B^-dependent genes as the result of a gene dosage shift has been demonstrated for C. difficile exposed to DNA polymerase inhibitors such as the phase II drug ibezapolstat/ACX-362E (20).

In this study, we demonstrate that σ^B^ overexpression is detectable and is tolerated for short periods of time. This allowed for the experimental identification of a set of genes that is most likely directly regulated by σ^B^ by performing transcriptome analyses under conditions of acute σ^B^ overexpression. The results obtained show that genes involved in the oxidative and nitrosative stress response form the core of the regulon. Additionally, we show that various antimicrobials and hydrogen peroxide induce the expression of σ^B^-regulated genes in a σ^B^-dependent manner, suggesting a link between the lethal exposure to antimicrobials and oxidative and nitrosative stresses in C. difficile.

## RESULTS

### C. difficile σ^B^ is measurably overproduced upon induction of the *sigB* gene.

Previous investigations of σ^B^ in C. difficile have used a *sigB* mutant and characterized its gene expression in the stationary growth phase in comparison with that of a wild-type strain (19). Although informative, this method is likely to result in indirect effects of σ^B^ due to stationary-phase heterogeneity, prolonged incubation, and possible positive or negative feedback in the σ^B^ regulatory circuit. To circumvent these issues and identify genes likely to be regulated by σ^B^ directly, we set out to uncouple *sigB* expression from its native regulatory circuit by expressing it from an inducible promoter.

**FIG 1:**
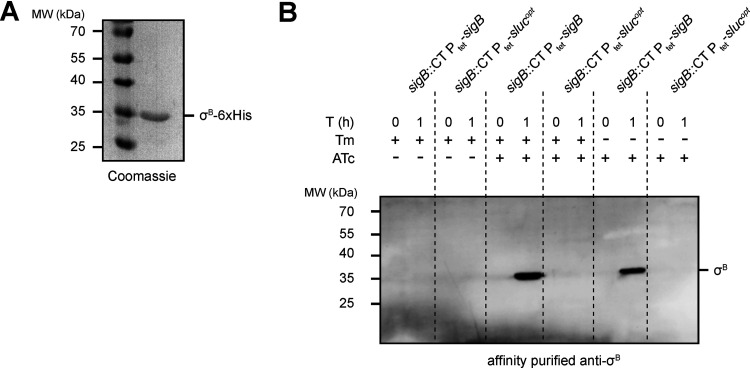
Recombinant σ^B^_6×His_ was used to generate a Clostridiodes difficile σ^B^-specific antibody for intracellular detection. (A) Coomassie blue-stained 12.5% SDS-PAGE gel of purified recombinant σ^B^_6×His_. (B) Western blot using affinity purified σ^B^ antibody (1:500) on strains IB58 (*sigB*::CT P_tet_-*sigB*) and IB61 (*sigB*::CT P_tet_-*sluc^opt^*). Cells were grown in lincomycin (20 μg/ml) and in the presence or absence of thiamphenicol (20 μg/ml) until an optical density at 600 nm (OD_600_) of ≈0.3, after which the indicated samples were induced with 100 ng/ml anhydrotetracycline (ATc). Samples were collected directly after the addition of ATc (or at the time ATc would have been added in the uninduced controls) at *T* = 0 h and after 1 h of induction (*T* = 1).

First, in order to confirm overproduction of σ^B^, we measured cellular σ^B^ levels using immunoblotting. For this purpose, we heterologously overproduced and purified σ^B^ containing a C-terminal His tag (Fig. 1A) and used this protein to raise a polyclonal antiserum. Corresponding polyclonal antibodies were affinity purified to prevent unspecific immune reactions.

Next, we set out to validate the overproduction of σ^B^ in transconjugant C. difficile cells harboring plasmids containing *sigB* under the control of the anhydrotetracycline (ATc)-dependent promoter P_tet_ (21). For this purpose, σ^B^ was produced in a *sigB* mutant background (strain IB58; *sigB*::CT P_tet_-*sigB*). As a control, we introduced a nonrelated expression construct in the same background (IB61; *sigB*::CT P_tet_-*sluc^opt^*) such that this control strain carries a plasmid with the same replicon, resistance marker, and inducible promoter.

We expected a signal at approximately 30 kDa in Western blot experiments for cells grown in the presence of the inducer ATc for strain IB58, but not for the uninduced cultures of IB58 or the control strain IB61. Additionally, by growing cultures in the presence or absence of thiamphenicol, we investigated whether overproduction of σ^B^ required selection for the P_tet_-*sigB* expression plasmid.

When strains were grown in brain heart infusion (BHI) broth supplemented with 0.5% (wt/vol) yeast-extract (BHIY) supplemented with 20 μg/ml lincomycin and induced for 1 h with or without 100 ng/ml ATc in the presence or absence of 20 μg/ml thiamphenicol, we did not detect any signal at the molecular weight expected for σ^B^ in the ATc-induced control samples (*sigB*::CT P_tet_-*sluc^opt^*) or in any of the uninduced samples (Fig. 1B). In contrast, after 1 h of induction, a clear band of the expected molecular weight of σ^B^ (≈30 kDa) was observed only in the IB58 (*sigB*::CT P_tet_-*sigB*) samples (Fig. 1B). Plasmid selection by inclusion of thiamphenicol in the growth medium did not influence σ^B^ overproduction in this time frame, which might have occurred as a tradeoff between σ^B^ overexpression and cellular toxicity (see further below).

We conclude that the affinity-purified rabbit anti-σ^B^ antibody is specific for σ^B^ and can be used for its detection in lysates of C. difficile. Furthermore, it is possible to uncouple *sigB* expression from its tight regulatory network by ATc-inducible overexpression for 1 h in *trans*.

### Prolonged overexpression of σ^B^ is lethal and leads to a loss of plasmids harboring P_tet_-*sigB*.

**FIG 2:**
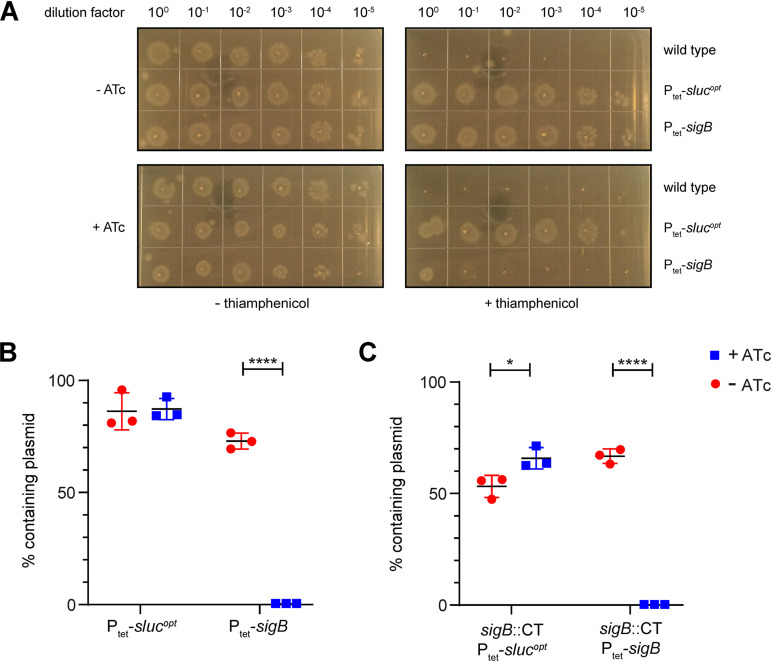
Overexpression of σ^B^ is toxic for C. difficile and leads to plasmid loss. (A) Tenfold serial dilutions on brain heart infusion broth supplemented with 0.5% (wt/vol) yeast-extract (BHIY) agar plates of the 630Δ*erm* (wild-type), AP34 (P_tet_-*sluc^opt^*), and JC096 (P_tet_-*sigB*) strains. Similar results were obtained for strains IB58 and IB61 (data not shown). (B) Percentages of cells retaining the plasmid in AP34 (P_tet_-*sluc^opt^*) and JC096 (P_tet_-*sigB*). (C) Percentages of cells retaining the plasmid in strains IB61 (*sigB*::CT P_tet_-*sluc^opt^*) and IB58 (*sigB*::CT P_tet_-*sigB*). Percentages were calculated based on the ratio of CFU/ml of the paired selective (with thiamphenicol) and nonselective (without thiamphenicol) plates. *, *P* < 0.05; ****, *P* ≤ 0.0001, as determined by an unpaired Student’s *t* test (*n* = 3).

Above, we showed that it is possible to overproduce σ^B^ in C. difficile and that this is tolerated by the bacterium for 1 h. This observation is somewhat at odds with the previously reported toxic nature of overproduced σ^B^ (8, 11). To reconcile these two observations, the effect of long-term overexpression of *sigB* and the stability of the plasmids used for σ^B^ overproduction under such conditions were investigated. First, overnight cultures of 630Δ*erm* (wild-type), AP34 (P_tet_-*sluc^opt^*), and JC096 (P_tet_-*sigB*) strains were adjusted for their optical density at 600 nm (OD_600_) values and 10-fold serially diluted. Subsequently, 2-μl spots per dilution were made on selective (20 μg/ml thiamphenicol) and nonselective BHIY agar plates, some of which contained 200 ng/ml ATc to induce P_tet_-dependent gene expression. All plates were then incubated anaerobically for 24 h. On plates without thiamphenicol, regardless of the presence of the inducer ATc, comparable growth was observed for all three strains (Fig. 2A). As expected, when selecting for the plasmid using thiamphenicol, no growth was observed for the susceptible 630Δ*erm* strain (which lacks the *catP* gene contained on the expression vector). In the absence of the inducer, no difference in growth was observed for the vector control strain (AP34; P_tet_-*sluc^opt^*) compared to the strain carrying the P_tet_-*sigB* plasmid (JC096). However, upon induction of *sigB* expression on selective plates, a 3- to 4-log growth defect was observed for the strain carrying P_tet_-*sigB* compared to the vector control strain. We conclude that prolonged induction of *sigB* expression is toxic when cells are cultured in the presence of thiamphenicol. Our results thus corroborate the finding that σ^B^ overproduction is toxic to C. difficile cells in liquid culture (11).

The lethality associated with σ^B^ overproduction was not seen when cells were grown without thiamphenicol in our experiment (Fig. 2A). We considered two possible explanations for this observation. As thiamphenicol is used for ensuring plasmid maintenance, its absence might result in plasmid loss due to segregation or negative selection pressure when a toxic protein such as σ^B^ is overproduced. The remaining cells that no longer express σ^B^ would consequently be susceptible to thiamphenicol (due to the loss of *catP*) but might outgrow those carrying the plasmid. Alternatively, the combination of σ^B^ and thiamphenicol might be toxic to the bacteria. To test whether plasmid loss was the cause of the observed lethality of bacteria overproducing σ^B^ in the presence of thiamphenicol, cells from the plates without thiamphenicol (with and without ATc) were resuspended in phosphate-buffered saline (PBS) at 1.0 McFarland turbidity and 10-fold serially diluted in brain heart infusion (BHI) medium. Spots (10 μl) of these dilutions were plated on plasmid-selective (thiamphenicol) and nonselective (no thiamphenicol) plates. Based on the ratio of CFU/ml of the selective and nonselective plates, the percentage of cells which lost their plasmid was calculated. If σ^B^ overproduction led to the loss of the plasmid under conditions that do not select for its maintenance (no thiamphenicol), we expected significantly reduced growth on plates containing thiamphenicol. Although some plasmid loss was observed under uninduced conditions, as well as for the negative-control strain AP34 (P_tet_-*sluc^opt^*), all cells originally containing the P_tet_-*sigB* plasmid (strain JC096) completely lost this plasmid upon induction of σ^B^ overproduction with ATc (Fig. 2B). Similar results were obtained for *sigB* mutant strains IB58 (*sigB*::CT P_tet_-*sigB*) and IB61 (*sigB*::CT P_tet_-sluc^opt^), indicating that the observed effects were solely due to in *trans* σ^B^ overproduction and did not result from an interference of the native *sigB* regulatory network (Fig. 2C). Together, these results are consistent with a model in which the vector with the low-copy-number pCD6 replicon is rapidly eradicated upon expression of a gene (here *sigB*) that causes lethal defects (22, 23).

### σ^B^ primarily activates genes relating to oxidative/nitrosative stress responses.

Above, we have shown that long-term overproduction σ^B^ is detrimental and that this leads to loss of the expression plasmid in the absence of thiamphenicol (Fig. 2), but that σ^B^ overproduction nevertheless could clearly be demonstrated when induction is limited to 1 h (Fig. 1C). Therefore, we used the time-limited induction to refine the previously proposed regulon (19) in both the presence and absence of thiamphenicol to strike a balance between potential secondary effects due to toxicity associated with σ^B^ overproduction (with thiamphenicol), and loss of the expression plasmid from a subpopulation of cells (without thiamphenicol) (Table 1). We compared transcriptome data from strain IB58 (*sigB*::CT P_tet_-*sigB*) to that of strain IB61 (*sigB*::CT P_tet_-*sluc^opt^*). IB61 harbors a vector for the inducible expression of a luciferase gene that does not lead to any toxicity or growth phenotype (24).

**TABLE 1:** Setup of the DNA array and numbers of differentially expressed genes, including numbers of positively and negatively regulated genes

Hybridization setup	Control	Target	Conditions	No. of genes^*a*^		
				DE	POS	NEG
1	*sigB*::CT P_tet_-*sluc^opt^* (IB61)	*sigB*::CT P_tet_-*sigB* (IB58)	Lincomycin (20 μg/ml), thiamphenicol (20 μg/ml), no ATc	5	4	1
2	*sigB*::CT P_tet_-*sluc^opt^* (IB61)	*sigB*::CT P_tet_-*sigB* (IB58)	Lincomycin (20 μg/ml), thiamphenicol (20 μg/ml), ATc (100 ng/ml)	183 (178)	167 (163)	16 (15)
3	*sigB*::CT P_tet_-*sluc^opt^* (IB61)	*sigB*::CT P_tet_-*sigB* (IB58)	Lincomycin (20 μg/ml), no thiamphenicol, no ATc	5	4	1
4	*sigB*::CT P_tet_-*sluc^opt^* (IB61)	*sigB*::CT P_tet_-*sigB* (IB58)	Lincomycin (20 μg/ml), no thiamphenicol, ATc (100 ng/ml)	150 (145)	136 (132)	14 (13)

a Numbers in brackets correspond to the number of differentially expressed genes after subtracting the differentially expressed genes identified in hybridizations 1 and 3 (that are not dependent on sigB induction). DE, differentially expressed; POS, positively regulated; NEG, negatively regulated.

We expected no genes or a limited number of genes to be differentially expressed (log_2_ fold change [log2FC] of ≤−1.5 or ≥1.5 and adjusted *P* value of <0.05) under noninducing conditions. Indeed, we found only five differentially expressed genes in the P_tet_-*sigB* strain (IB58) compared to the P_tet_-*sluc^opt^* control (IB61) strain (hybridizations 1 and 3) (see Data Set S1 in the supplemental material). These genes were similarly positively (CD0583 and CD0584, both GGDEF domain-containing proteins [25], and CD2214 and CD2215, both potential transcriptional regulators [26]) and negatively (CD1616, an EAL domain protein [25]) regulated in all hybridizations, including those where *sigB* expression was not induced. These results suggest that the basis for the observed differential expression of these genes was vector specific but not dependent on σ^B^ induction. These genes were therefore not investigated further and are excluded from the numbers discussed below.

10.1128/mSphere.00728-20.1 DATA SET S1 Genes differentially expressed in σ^B^-overproducing cells compared to controls. Cells were harvested from cultures with (induced) or without (uninduced) anhydrotetracycline (ATc), and with (+Tm) or without (−Tm) thiamphenicol. Gene name is the generic gene name (or locus tag if a gene name is not available). log_2_FC is the log_2_ of the fold change in gene expression. Four different comparisons are shown, and genes are aligned between comparisons. Upregulated genes are indicated in green. Downregulated genes are indicated in red. Genes not considered not part of the σ^B^ regulon are highlighted in yellow. Download Data Set S1, XLSX file, 0.04 MB. Copyright © 2020 Boekhoud et al. 2020 Boekhoud et al. This content is distributed under the terms of the Creative Commons Attribution 4.0 International license.

**FIG 3:**
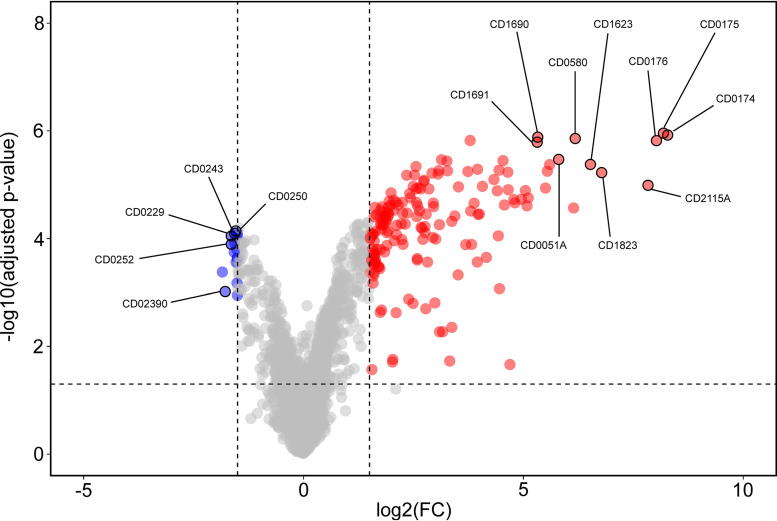
Volcano plot of the transcriptome analysis of the σ^B^ regulon. Graphical representation of differential gene regulation upon overproduction of σ^B^. Dashed lines indicated the following significance threshold: |log2FC| of >1.5 and adjusted *P* value of <0.05. Genes significantly upregulated by σ^B^ are indicated in red, and downregulated genes are indicated in blue. The top 10 upregulated genes and 5 selected downregulated genes are annotated in the figure. An interactive version of the graph is available for exploration via the URL provided in Text S1 in the supplemental material.

Upon induction of *sigB* expression, 145 genes were differentially expressed when strains were cultured without thiamphenicol (hybridization 4), and 178 genes were differentially expressed when thiamphenicol was present during cultivation (hybridization 2) (Fig. 3 and Table 1 and Data Set S1). The majority showed an increase in expression upon induction of *sigB* expression (132 in the samples without thiamphenicol and 163 in the samples with thiamphenicol), consistent with its function as a sigma factor (27), while a minority revealed a decreased expression (13 in the samples without thiamphenicol and 15 in the samples with thiamphenicol). Of note, we observed only a minor difference in the number of differentially expressed genes between the cells grown in the absence and presence of thiamphenicol (33 genes).

10.1128/mSphere.00728-20.3 TEXT S1 Link to access an interactive version of the volcano plot based on hybridization 2 (cells harvested from cultures grown in the presence of both anhydrotetracycline and thiamphenicol). Download Text S1, PDF file, 0.05 MB. Copyright © 2020 Boekhoud et al. 2020 Boekhoud et al. This content is distributed under the terms of the Creative Commons Attribution 4.0 International license.

Together, these results demonstrate a high level of consistency in the σ^B^ regulon despite potential plasmid loss (when grown in the absence of thiamphenicol) or toxic effects (when grown in the presence thiamphenicol). Our results also show that σ^B^ primarily activates gene expression.

We focused our further analyses on the data obtained from hybridization 2 (with ATc and thiamphenicol), as this condition provided the broadest data set (178 differentially expressed genes) for the redefinition of the σ^B^ regulon under our experimental conditions (Data Set S1).

Of the 163 genes upregulated by σ^B^, the vast majority appeared to be associated with an response to oxidative stress, since they encode various oxidoreductases, peroxidases, and thioredoxin reductases (Table 2). Notably, approximately 51% of the 98 genes previously found to be upregulated under aerobic stress (7) were also positively regulated by σ^B^ (Table 2). Five additional genes associated with aerobic/nitrosative stress (*cooS* [*cd0174*], *iscS2* [*cd1279*], the *cd1280* gene, *cysK* [*cd1594*], the *cd1823* gene, and *msrAB* [*cd2166*]) were also found to be induced by σ^B^, in agreement with previous findings (19).

**TABLE 2:** Selected genes differentially expressed upon overexpression of σ^B^^*a*^

Gene group or locus tag^*b*^	Gene name	log_2_FC	Adjusted *P* value	Predicted function
Genes upregulated by aerobic stress and positively regulated by σ^B^				
CD630DERM_00530	*mrnC*	1.9	3.29E-05	Ribonuclease III domain
CD630DERM_01750		8.2	1.11E-06	Oxidoreductase, Fe-S subunit
CD630DERM_01760		8.0	1.52E-06	Oxidoreductase, NAD/flavin adenine dinucleotide (FAD) binding subunit
CD630DERM_01920	*cls*	2.6	2.35E-04	Cardiolipin synthetase 1
CD630DERM_03500		1.9	4.63E-05	Putative hydrolase, HAD superfamily
CD630DERM_03510		1.8	5.66E-05	Conserved hypothetical protein
CD630DERM_05600	*nfo*	4.0	3.5E-05	Endonuclease IV
CD630DERM_05610		2.0	2.39E-05	Putative aldo-/ketoreductase; putative ferredoxin
CD630DERM_05650	*nth*	3.7	5.66E-06	Endonuclease III
CD630DERM_05660		2.5	6.62E-06	Putative tRNA/rRNA methyltransferase
CD630DERM_05800	*gapN*	6.2	1.38E-06	Glyceraldehyde-3-phosphate dehydrogenase (NADP^+^) (GAPDH)
CD630DERM_11250		5.5	1.15E-05	Nitroreductase family protein
CD630DERM_11570	*norV*	4.0	5.16E-06	Anaerobic nitric oxide reductase flavorubredoxin (FlRd) (FlavoRb)
CD630DERM_13410	*exoA*	1.8	6.76E-05	Exodeoxyribonuclease
CD630DERM_14630		1.8	1.77E-04	Conserved hypothetical protein
CD630DERM_15240		4.8	1.88E-05	Putative rubrerythrin
CD630DERM_15260	*pyrC*	1.6	1.98E-04	Dihydroorotase
CD630DERM_15760		1.6	2.71E-02	Putative arylesterase
CD630DERM_16220		2.7	1.03E-04	Putative lipoprotein
CD630DERM_16230		6.5	4.21E-06	NADH-oxygen oxidoreductase
CD630DERM_16240	*vanR*	1.6	8.84E-05	Two-component response regulator
CD630DERM_16900	*trxA*	5.3	1.31E-06	Thioredoxin reductase
CD630DERM_16910	*trxB*	5.3	1.62E-06	Putative thioredoxin disulfide reductase
CD630DERM_17780		2.6	1.19E-05	Conserved hypothetical protein
CD630DERM_17790		2.4	1.46E-05	Conserved hypothetical protein
CD630DERM_18180	*ispH*	1.8	5.35E-05	4-Hydroxy-3-methylbut-2-enyl diphosphate reductase
CD630DERM_18220	*bcp*	6.1	2.70E-05	Putative thiol peroxidase
CD630DERM_18970		1.8	3.20E-05	Conserved hypothetical protein
CD630DERM_19430		3.3	3.62E-06	Conserved hypothetical protein
CD630DERM_20460		5.5	5.60E-06	Conserved hypothetical protein
CD630DERM_21170	*trxB2*	5.0	1.86E-05	Thioredoxin reductase
CD630DERM_21650		2.2	1.04E-04	Transcriptional regulator, helix-turn-helix (HTH)-type
CD630DERM_24760		4.6	5.84E-06	Conserved hypothetical protein
CD630DERM_27960	*cwp10*	5.1	2.47E-05	Cell surface protein
CD630DERM_27970		5.1	1.75E-05	Putative calcium binding adhesion protein
CD630DERM_29930		3.8	3.07E-05	Conserved hypothetical protein
CD630DERM_30380		3.9	2.21E-05	Conserved hypothetical protein
CD630DERM_30390		4.1	1.07E-05	Putative ATPase
CD630DERM_30400		2.1	8.46E-05	Conserved hypothetical protein
CD630DERM_30420		2.6	2.47E-05	Putative membrane protein
CD630DERM_33070		3.8	1.51E-06	Putative phosphoesterase
CD630DERM_33100		3.1	6.31E-06	Putative d-isomer specific 2-hydroxyacid dehydrogenase
CD630DERM_33110		2.9	1.19E-05	Conserved hypothetical protein
CD630DERM_34080		2.3	3.38E-05	Putative DNA mismatch repair ATPase MutS
CD630DERM_34090	*scoC* (*hprK*)	2.4	1.74E-05	Phosphotransferase (PTS) system, HPr kinase/phosphorylase
CD630DERM_34100	*uvrC*	2.7	1.19E-05	Excinuclease subunit C
CD630DERM_34730	*atpC2*	1.6	2.90E-04	ATP synthase C chain
CD630DERM_34760	*atpZ*	1.6	2.42E-04	Putative ATP synthase protein
CD630DERM_36100		4.6	2.38E-05	Conserved hypothetical protein
CD630DERM_36140		2.5	1.07E-05	Conserved hypothetical protein, DUF1130 family
Other genes involved in oxidative/nitrosative stress positively regulated by σ^B^				
CD630DERM_01740	*cooS*	8.3	1.20E-06	Carbon monoxide dehydrogenase
CD630DERM_12790	*iscS2*	2.8	2.00E-03	Cysteine desulfurase
CD630DERM_12800		3.0	1.56E-03	Putative NifU-like protein
CD630DERM_14740		5.0	1.28E-05	Putative rubrerythrin (Rr)
CD630DERM_15940	*cysK*	3.4	4.44E-03	*O*-acetyl-serine thiol-lyase A (*O*-acetyl-sulfhydrylase) (OAS-TL)
CD630DERM_18230		6.8	5.93E-06	Conserved hypothetical protein, UPF0246 family
CD630DERM_21660	*msrAB*	4.7	1.24E-05	Peptide methionine sulfoxide reductase MsrA/MsrB

a Genes positively regulated by σ^B^ and aerobic stress and other genes involved in oxidative/nitrosative stress (7, 19) are highlighted here.

b CD numbers corresponding to the published annotation of strain CD630 (52) can be derived by removing “630DERM” and removing the last digit (in case of a 0) or replacing it with an A (in the case of a 1).

Our findings are recapitulated in a volcano plot (28), which clearly shows that genes with lower expression upon *sigB* induction (in blue) cluster close to the significance threshold, whereas those with increased expression (in red) show a larger fold change (Fig. 3). We calculated the Manhattan distance for each data point (see Data Set S2 in the supplemental material), and discuss the proteins encoded by the top 10 differentially expressed genes below.

10.1128/mSphere.00728-20.2 DATA SET S2 Manhattan distance for differentially regulated genes from hybridization 2 (cells harvested from cultures grown in the presence of both ATc and thiamphenicol). Change indicates whether expression is higher (increased) or lower (decreased) upon overproduction of σ^B^. log_2_FC represents log2 of the fold change in gene expression. Significance is given as −10 · log of the adjusted *P* value (see Data Set S1). Manhattan distance was calculated by and exported using the online tool VolcaNoseR. Download Data Set S2, XLSX file, 0.02 MB. Copyright © 2020 Boekhoud et al. 2020 Boekhoud et al. This content is distributed under the terms of the Creative Commons Attribution 4.0 International license.

CD0051A is a small hypothetical protein of unknown function. It does not contain any recognizable domains, and a secondary structure prediction using Phyre2 does not give any clues as to its potential function (29). CD0580 (GapN) is annotated as a glyceraldehyde-3-phosphate dehydrogenase (GAPDH), a key glycolytic enzyme, and contains an aldehyde dehydrogenase domain. Interestingly, its activity has been shown to be redox controlled in other bacteria and has been implicated in the response to reactive oxygen and nitrogen species (30–32). CD1623 is a putative oxidoreductase with similarity to FAD flavoproteins and rubredoxins. CD1690 (TrxA) and CD1691 (TrxB) are likely encoded in the same operon (33) and form a thioredoxin/thioredoxin-disulfide reductase couple. CD0174 (CooS; InterPro family IPR010047), CD0175, and CD0176 are likely also encoded in a single operon (33) and function as carbon monoxide dehydrogenase and two putative oxidoreductases. As mentioned above, CD0174 has been implicated in aerobic/nitrosative stress, and it is likely that CD1623, CD1690, and CD1691 also function in this pathway. Finally, CD2115A encodes another small hypothetical protein; as for CD0051A, no function could be assigned on the basis of secondary structure prediction.

As the σ^B^ regulon that we define here is substantially smaller than that previously reported, the major conclusion is that at least 32% of the σ^B^ regulon is involved in positively regulating oxidative/nitrosative stress responses. In the previous investigation of the σ^B^ regulon they were approximately 3.2% (≈32/1,000) (19). Overall, we conclude that the core functions of the σ^B^ regulon lie in the regulation of the detoxification response to oxygen and nitro radicals.

### *In vitro* runoff transcriptions demonstrate direction activation of P*_cd0350_*, P*_cd2963_*, P*_cd3412_*, and P*_cd3605_* by σ^B^.

Gene expression can directly or indirectly be influenced by σ^B^, and to date no attempts have been made to discriminate these possibilities biochemically (11, 19). Despite the short time of induction and the uncoupling of σ^B^ from its normal regulatory network, our analyses could possibly also have picked up indirect effects. To determine if the transcription of selected genes is directly activated by σ^B^, *in vitro* transcription runoff reactions were performed using purified σ^B^_6×His_ and RNA polymerase core enzyme (RNAP^core^) on the upstream regions of a selection of genes. The genes *cd0350* (encoding a putative hydrolase involved in oxidative stress; Table 2), *cd2963* (encoding an l,d-transpeptidase), *cd3412* (encoding UvrB, involved in nucleotide excision repair), *cd3605* (encoding a ferredoxin), and *cd3614* (encoding a hypothetical protein involved in oxidative stress; Table 2) were selected on the basis of differential expression in our transcriptome analyses (Data Set S1) and those of others (19), availability of reporter constructs that could be used to generate a template for the *in vitro* transcription reactions (20), and/or the presence of a putative σ^B^ consensus upstream in the upstream region (11). The gene *cd0872* (encoding maltose *O*-acetyltransferase) was not differentially expressed in our transcriptome data and was thus included as a negative control. The promoter of the toxin A gene (*tcdA*) in combination with purified TcdR was used as a positive control for the assay, as previously described (34).

**FIG 4:**
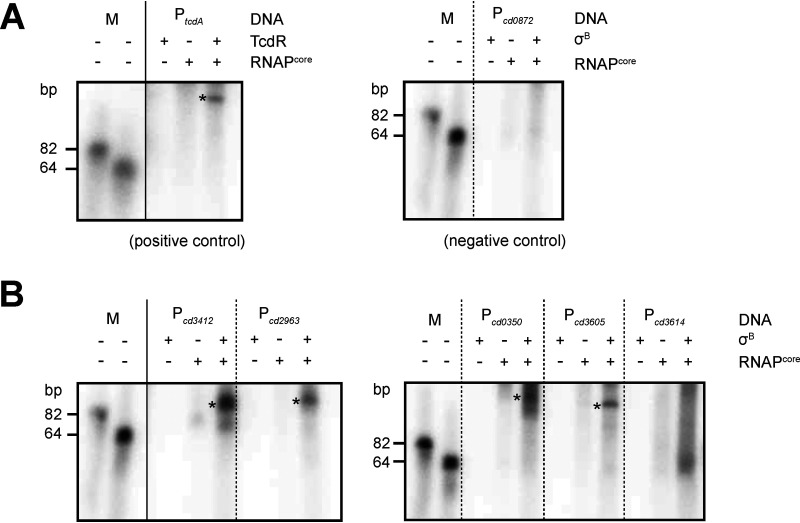
*In vitro* runoff transcription of selected promoter regions. Samples were run on an 8% urea gel. The two bands corresponding to 82 bp and 64 bp are end-labeled oligonucleotides. Reactions without sigma factor (σ^B^ or TcdR, respectively) or RNAP^core^ were analyzed as controls. Asterisks indicate the presence of distinct transcripts. RNAP^core^, Escherichia coli RNA polymerase core enzyme (NEB). (A) Controls for the *in vitro* runoff transcriptions. Purified TcdR, a sigma factor demonstrated to activate *tcdA* transcription *in vitro* (34), was used with P*_tcdA_* (from plasmid pCD22) as a positive control for the assay. P*_cd0872_* (derived from pIB21) shows no altered transcription in the DNA array analysis and was taken along as a negative control. (B) *In vitro* runoff transcriptions for selected genes induced by σ^B^ overproduction.

As expected, no *in vitro* transcript was observed for a linear DNA fragment containing P*_cd0872_* incubated with purified σ^B^_6×His_ and RNAP^core^ under our experimental conditions, whereas a specific product was obtained for the positive control P*_tcdA_* in the presence of TcdR and RNAP^core^ (Fig. 4). An RNAP^core^- and σ^B^_6×His_-specific signal was observed for fragments containing the putative promoter regions of the genes *cd0350*, *cd2963*, *cd3412*, and *cd3605*, demonstrating that expression of these genes was directed by σ^B^. For the fragments containing the putative promoter of *cd3614*, we did not get a consistent product in the *in vitro* transcription experiments, although some smearing is visible in the lane with RNAP^core^ and σ^B^_6×His_. As *cd3614* demonstrates clear differential expression in the DNA array experiments and it upstream region harbors the σ^B^ consensus sequence WGWTT-N_13-17_-(G/T)GGTWA (19), we consider it likely that this gene is directly regulated by σ^B^ and that our failure to obtain a discrete signal is due to our experimental conditions or to the lack of an auxiliary factor in our *in vitro* assays.

Overall, we provide the first biochemical evidence for direct σ^B^-dependent activation of several genes identified via transcriptome analyses as part of the σ^B^ regulon in C. difficile.

### Antimicrobials and hydrogen peroxide activate σ^B^-directed gene transcription.

**FIG 5:**
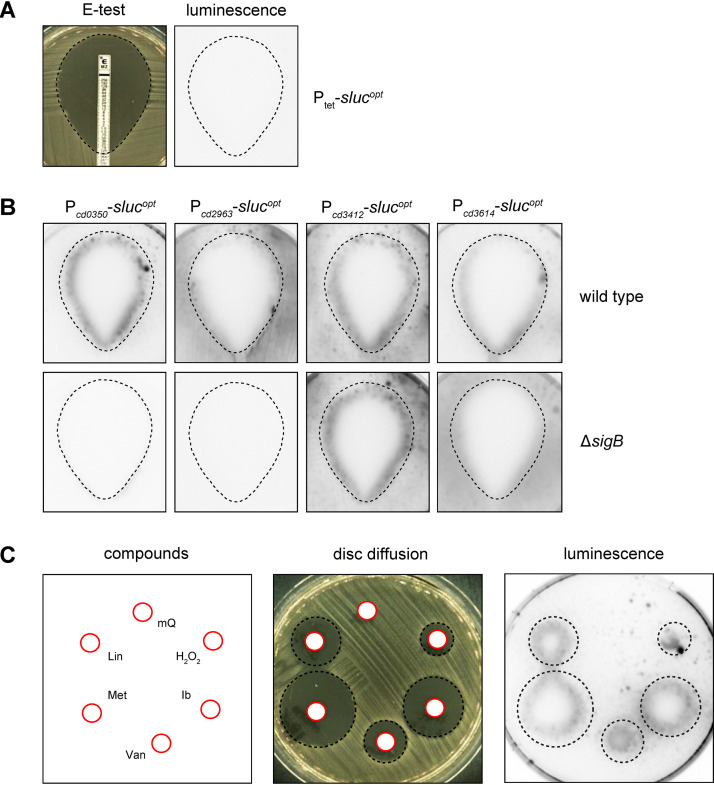
Plate-based luciferase assay shows σ^B^-dependent promoter activity from antimicrobial and hydrogen peroxide exposure. (A) Setup of the assay. Etest halos (left) were sprayed with luciferase substrate and imaged (right). The dotted line indicates the location of the halo based on the left panel. Strain AP34 (P_tet_-*sluc*^opt^) shows no signal due to the absence of inducer. (B) Luciferase reporters of different σ^B^-regulated promoters were tested for luciferase signal after a metronidazole Etest. Halos are indicated by the dashed lines as in panel A. (C) Luciferase activity of the P*_cd0350_* luciferase reporter was imaged in the presence of discs containing 10 μl of the following compounds: sterile H_2_O (mQ), lincomycin (Lin; 3,000 μg/ml), metronidazole (Met; 200 μg/ml), vancomycin (Van; 200 μg/ml), ibezapolstat (Ib; 400 μg/ml) and hydrogen peroxide (H_2_O_2_; 1 M). Halos and the location of the different stressors are indicated by red circles (disc) and black dashed circles (halo). Signals outside the halo in these images are representative for the signals across the plate. Experiments were performed at least in triplicate with qualitatively similar results.

The redefined σ^B^ regulon points toward a substantial role for σ^B^ in coordinating the oxidative and nitrosative stress response, which could result from antimicrobial treatment. In order to test for the activation of σ^B^-dependent promoters by antimicrobials, we set up a plate-based luciferase reporter assay. In this assay, cells harboring σ^B^-dependent luciferase reporter constructs were plated on BHIY agar to give confluent growth and exposed to antimicrobials either through an epsilometer test (Etest) or through a filter disc. Subsequently, luciferase activity was imaged (for details, see Materials and Methods). A strain harboring P_tet_-*sluc^opt^* (AP34) served as negative control, as this promoter is not expected to respond in a σ^B^-dependent manner (Fig. 5A).

First, the σ^B^-dependent response to metronidazole was investigated. Metronidazole, formerly used as a first-line treatment for CDI, is believed to cause DNA damage through the formation of nitro radicals, although its exact mode of action remains unclear (6, 35). To survey a full spectrum of metronidazole concentrations, we evaluated luminescence after 24-h incubation of a metronidazole Etest. If metronidazole treatment results in σ^B^-dependent activation of gene transcription, we expect to see a luciferase signal in the wild type but not in a σ^B^ knockout background. In agreement with this, activation of P*_cd0350_* was observed at the edge of the halo resulting from the metronidazole Etest in the wild-type background but not in the σ^B^ knockout strain (Fig. 5B). No signal was observed for the negative control P_tet_-*sluc^opt^* (Fig. 5A). The observed σ^B^-dependent activation of gene expression at the edge of the halo but not further into the plate suggests that the metronidazole-induced, σ^B^-dependent activation of P*_cd0350_* occurred close to the MIC. Expression of the luciferase from P*_cd296_*_3_ was found to be strictly dependent on σ^B^, as no luciferase activity was observed in the *sigB* knockout strain. However, there was limited to no increase in reporter gene expression in the presence of metronidazole. Metronidazole strongly activated transcription from P*_cd3412_* at MIC levels of metronidazole, but this appeared to be independent of σ^B^ in this assay since in the *sigB* mutant a similar induction was observed. Finally, in a manner comparable to that of P*_cd0350_*, the activation of P_CD3614_ was strongly induced by metronidazole at values close to the MIC in a σ^B^-dependent manner, but residual activity was observed in the σ^B^ knockout strain independent of metronidazole levels. We noted that metronidazole-induced promoter activation appeared to occur on the inside of the Etest halo, which might be attributed to the secretion of the luciferase reporter.

The observed diverse regulatory responses at different tested promoters during the treatment of C. difficile with metronidazole (with respect to basal level, *sigB* dependence, and induction) pointed toward a more complex regulatory network with the participation of σ^B^ but also influenced by other factors. Antimicrobial-driven (and σ^B^-dependent) activation of σ^B^ target genes could be specific to metronidazole or represent a more general response to cellular (toxic) stresses. Therefore, we evaluated the effects of different antimicrobial compounds and the radical producer H_2_O_2_ as a positive control (19), using the P*_cd0350_* reporter construct, as this promoter demonstrated the clearest σ^B^-dependent activation in the presence of metronidazole (Fig. 5B). We tested the cell wall biosynthesis inhibitor vancomycin, the protein synthesis inhibitor lincomycin, and the DNA polymerase inhibitor ibezapolstat (formerly known as ACX-362E) (20). We observed clear activation of P*_cd0350_* in the presence of all added stressors but not for a negative control containing water (Fig. 5C).

We conclude that, at least for the σ^B^-dependent promoter of *cd0350*, activation does not only occur upon exposure to lethal levels of metronidazole but also occurs with unrelated antimicrobials and toxic stressors such as hydrogen peroxide.

## DISCUSSION

In this work, we have demonstrated by Western blotting using an affinity-purified anti-σ^B^ antibody that σ^B^ can be overproduced for a limited period of time, sufficient for transcriptome analyses. The induced production of σ^B^ in a *sigB* mutant background yielded highly consistent results despite potential toxicity and plasmid loss (Fig. 2), and the results were used to redefine the surprisingly large σ^B^ regulon previously proposed (19). As our approach more accurately measures changes in transcription directly related to σ^B^ production, the refined regulon described here is much smaller (see Data Set S1 in the supplemental material). Its size is fully in line with that of the σ^B^ regulon of other Gram-positive bacteria such as L. monocytogenes (≈130 genes), B. subtilis (≈150 genes), and S. aureus (≈200 genes) (8). The redefined regulon underscores the importance of σ^B^ in responding to oxidative/nitrosative stresses, as genes implicated in such processes are significantly enriched in the smaller regulon.

The majority of the genes in our regulon were found to be induced, rather than repressed, by σ^B^. This is in line with sigma factors acting as specificity determinants for transcription initiation (27). Similar observations have been made for the σ^B^ regulon of L. monocytogenes (36, 37). For the first time, direct evidence of C. difficile σ^B^-dependent gene activation is provided by the results of the *in vitro* runoff transcriptions (Fig. 4), which demonstrate that RNAP^core^ and σ^B^ are sufficient to generate transcripts from P_*cd0350*_, P_*cd2963*_, P_*cd3412*_, and P_*cd3605*_. Notably, these experiments pave the way for a further *in vitro* characterization of this sigma factor in C. difficile, including validation of the σ^B^ binding sequence and the interplay with other regulators.

Although the promoters of *cd3412* (*uvrB*) and *cd3614* were reported to have a σ^B^ consensus sequence and are differentially expressed upon σ^B^ overexpression (19, 20), our results clearly demonstrate that they can also be expressed in a σ^B^-independent manner (Fig. 5B). This is most notable for P*_cd3412_*, which is still activated by metronidazole in the absence of σ^B^, in line with results obtained with ibezapolstat in a different study (20). Both metronidazole and ibezapolstat treatment can cause DNA damage, and DNA-damage dependent induction of *cd3412* therefore likely depends on a *sigB*-independent pathway.

The observed σ^B^-dependent gene repression is expected to be indirect (σ^B^ induces the transcription of a repressor gene), or the result of competition (σ^B^ competes with other sigma factors for RNAP), as sigma factors by their very nature induce gene expression (27). We consider the second scenario more likely for the following reasons. First, little overproduction of σ^B^ was detected after 30 min of induction. This leaves only a limited time for indirect effects to occur in our setup. Second, the majority of genes downregulated upon overexpression of σ^B^ fall into a single functional group (flagellar motility). These genes are known to be regulated by the dedicated sigma factor, σ^D^ (38), supporting the model of sigma factor competition. Strikingly, in L. monocytogenes, σ^B^ activity (indirectly) also results in downregulation of flagellar gene expression, but this is mediated by the repressor MogR (39). Protein BLAST analyses revealed that C. difficile does not possess a MogR homologue. Nevertheless, the conserved inverse correlation between the σ^B^-dependent general stress response and bacterial motility could represent a cost-saving strategy for bacterial cells (40). The indirect mechanism underlying the observed σ^B^-dependent downregulation in C. difficile remains to be determined.

There appears to be an intriguing link between σ^B^ and the response to toxic compounds, as a *sigB* mutant was more susceptible to rifampin and mitomycin C (19), and exposure to antimicrobials (metronidazole, vancomycin, lincomycin, and ibezapolstat) or hydrogen peroxide leads to σ^B^-dependent promoter activation (Fig. 5C). The mechanism behind the latter is unclear. It has been suggested that antimicrobials at toxic concentrations can influence metabolism and respiration (41, 42), potentially resulting in the formation of bactericidal concentrations of radical species (43–45). A strong connection between σ^B^ and oxidative (and/or nitrosative) stress in C. difficile (Table 2) and other bacteria (7, 18, 19), as well as a recently described radical scavenging strategy that increases tolerance to antimicrobials (46), are consistent with such a model. However, additional research is necessary to determine exactly how these processes occur and are influenced by antimicrobials in anaerobic organisms under anoxic conditions.

In conclusion, we have demonstrated that σ^B^ is directly involved in metabolic and oxidative stress responses and that lethal stresses may influence these processes, resulting in activation of σ^B^-targeted genes.

## MATERIALS AND METHODS

### Construction of σ^B^ expression and luciferase reporter vectors.

All oligonucleotides used in this study can be found in Table 3. Plasmids and strains are listed in Table 4. All PCR products used for sequencing or plasmid synthesis were generated with Q5 high-fidelity polymerase (NEB). The P_T7_-*sigB*_6×His_ expression vector pIB14 was created by restriction-ligation using the restriction enzymes NdeI and XhoI. Using primers oIB-1 and oIB-2 on C. difficile 630Δ*erm* chromosomal DNA, the *sigB* coding sequence (CDS) was amplified by PCR. The resulting DNA fragment was digested and ligated into NdeI-XhoI-digested pET21b(−) vector, generating expression vector pIB14. Plasmids pIB27, pIB68, pIB69, and pIB74 have been described previously (20). The *cd0872* promoter area was amplified using primers oIB-14 and oIB-15, and the P*_cd0872_* luciferase-reporter plasmid was created by restriction-ligation using restriction enzymes KpnI and SacI in digested pAP24 backbone, generating plasmid pIB21. The P*_cd3605_* luciferase reporter plasmid was generated by Gibson assembly as described previously (20) using primers oIB-90 and oIB-99, yielding plasmid pIB73. A plasmid containing P_tet_-*sigB* was generated by cloning the 630Δ*erm sigB* CDS amplified with oWKS-1498 and oWKS-1499 in pMiniT (catalog no. E1202; NEB) per the manufacturer’s instructions. Using restriction enzymes SacI and BamHI, this PCR fragment was cloned into pRPF185, yielding pWKS1760. All plasmids were verified by Sanger sequencing.

**TABLE 3:** Oligonucleotides used in this study

Name	Sequence (5′–3′)^*a*^	Description	Source or reference
Cdi-sigB-F	GTAGCTAATGCTACACATTAC	Verification of *sigB* ClosTron mutant	This study
Cdi-sigB-R	CAGTCATCTGTGATATCCCTAG	Verification of *sigB* ClosTron mutant	This study
EBSuniversal	CGAAATTAGAAACTTGCGTTCAGTAAA	Verification of *sigB* ClosTron mutant	49
ErmRAM-F	ACGCGTTATATTGATAAAAATAATAGTGGG	Verification of *sigB* ClosTron mutant	49
ErmRAM-R	ACGCGTGCGACTCATAGAATTATTTCCTCCCG	Verification of *sigB* ClosTron mutant	49
oIB-1	TAGCCATATGAAAAATGTAGCTAATGCTACAC	Forward primer for *sigB* cloning in pET21b, contains a NdeI restriction site	This study
oIB-2	ACTGCTCGAGTAAATTTTTTTCATATTCTTTTTTCAG	Reverse primer for *sigB* cloning in pET21b, contains an XhoI restriction site	This study
oIB-14	GTACGGTACCTTTACATATACTATATATGTTAGAAAAAC	Forward primer for P*_cd0872_* containing a KpnI restriction site	This study
oIB-15	GGTAGAGCTCATAGTTTACTCCTTTTTGTTATAATTG	Reverse primer for P*_cd0872_* containing a SacI restriction site	This study
oIB-26	GGAAGGTACCGTTGAATAAAGTATTTATTTTCCATG	Forward primer for P*_cd3412_* containing a KpnI restriction site	20
oIB-27	GGTAGAGCTCAGTATCACTCCTTTTTTCGAAC	Reverse primer for P*_cd3412_* containing a SacI restriction site	20
oIB-80	ctagcataaaaataagaagcctgcatttgcAAATTTACGAAAAGCTTGC	Forward primer for P*_cd0350_*	20
oIB-82	ctagcataaaaataagaagcctgcatttgcTTGTGTTTAAGGGATTTTGAAAG	Forward primer for P*_cd2963_*	20
oIB-90	ctagcataaaaataagaagcctgcatttgcATGTAAAGAAGCCGAAGAAG	Forward primer for P*_cd3605_*	This study
oIB-92	ctagcataaaaataagaagcctgcatttgcGAATAAAAAAGGTGGTGTC	Forward primer for P*_cd3614_*	20
oIB-94	agctattaataattttttacttggtctcatTTTTACCTCCATGTAACATTTATTG	Reverse primer for P*_cd0350_*	20
oIB-95	agctattaataattttttacttggtctcatAATTAAATCCTTCCTTACATTGTAATTAC	Reverse primer for P*_cd2963_*	20
oIB-99	agctattaataattttttacttggtctcatATTTCAGCCCTCCATATTTG	Reverse primer for P*_cd3605_*	This study
oIB-100	agctattaataattttttacttggtctcatATAAACACCCTCCTATTCTTTG	Reverse primer for P*_cd3614_*	20
oWKS-1498	GAGCTCCTGCAGTAAAGGAGAAAATTTTATGAAAAATGTAGCTAATGCTACAC	Forward primer for *sigB* CDS	This study
oWKS-1499	GGATCCTTATAAATTTTTTTCATATTCTTTTTTCAG	Reverse primer for *sigB* CDS	This study
oWKS-1513	GAGCTCAAATTTGAATTTTTTAGGGGGAAAATACCATGCATCATCACCATCACCACGGTTCCGAAATCGGTACTGGCTTTCC	Oligonucleotide used for end labeling	This study
oWKS-1506	GAGCTCAAATTTGAATTTTTTAGGGGGAAAATACCATGGTTTCAAAAGGAGAAGAATTATTTAC	Oligonucleotide used for end labeling	This study

a Restriction sites are underlined, and 30-bp overlapping regions used in Gibson Assembly are indicated in lowercase letters.

**TABLE 4:** Plasmids and strains used in this study

Name of plasmid or strain	Relevant features^*a*^	Source or reference
Plasmids		
pAP24	P_tet_-*sluc^opt^*; *catP*	24
pCD22	P_tcdA_; *catP*	53
pIB14	P*_T7_*-*sigB*_6×His_; *amp*	This study
pIB21	P*_cd0872_*-*sluc^opt^*; *catP*	This study
pIB27	P*_cd3412_*-*sluc^opt^*; *catP*	20
pIB68	P*_cd0350_*-*sluc^opt^*; *catP*	20
pIB69	P*_cd2963_*-*sluc^opt^*; *catP*	20
pIB73	P*_cd3605_*-*sluc^opt^*; *catP*	This study
pIB74	P*_cd3614_*-*sluc^opt^*; *catP*	20
pMTL007C-E2_sigB171s::intron_ermB	*sigB*-retargeted intron (*ermB*)	This study
pWKS1750	P_tet_-*sigB*; *catP*	This study
Strains		
630Δ*erm*	MLS-susceptible derivative of *Clostridioides difficile* strain 630	54, 55
AP34	630Δ*erm* pAP24; Thia^r^	24
DH5α	*Escherichia coli* F^−^ *endA1 glnV44 thi-1 recA1 relA1 gyrA96 deoR nupG purB20* ϕ*80dlacZ*ΔM15 Δ(*lacZYA-argF*)*U169 hsdR17*(*r_K_*^−^ *m_K_*^+^) λ^−^	Laboratory stock
IB14	Rosetta (DE3) pLysS pIB14; Amp^r^, Chlor^r^	This study
IB18	630Δ*erm sigB*::*CT*; Erm^r^/Linco^r^	This study
IB37	630Δ*erm* pIB27; Thia^r^	20
IB56	630Δ*erm* Δ*sigB*	20
IB58	IB18 pWKS1750; Thia^r^, Erm^r^/Linco^r^	This study
IB61	IB18 pAP24; Thia^r^, Erm^r^/Linco^r^	This study
IB95	630Δ*erm* pIB68; Thia^r^	20
IB96	630Δ*erm* pIB69; Thia^r^	20
IB98	IB56 pIB27; Thia^r^	20
IB99	IB56 pIB68; Thia^r^	20
IB100	IB56 pIB69; Thia^r^	20
IB108	630Δ*erm* pIB74; Thia^r^	20
IB111	IB56 pIB74; Thia^r^	20
JC096	630Δ*erm* pWKS1750; Thia^r^	This study

a MLS, macrolides-lincosamides-streptogramin B; Amp, ampicillin; Chlor, chloramphenicol; Erm, erythromycin; Linco, lincomycin; Thia, thiamphenicol; ^r^, resistance.

### Bacterial strains and growth conditions.

Strains of Escherichia coli were grown aerobically at 37°C in Luria-Bertani broth (Affymetrix) supplemented with ampicillin (50 μg/ml), kanamycin (50 μg/ml), and/or chloramphenicol (20 μg/ml) when required. Plasmids were maintained in E. coli strains DH5α or MDS42 (Scarab Genomics) under appropriate antimicrobial selection, and cells were transformed using standard procedures (47). For plasmid conjugation into recipient wild-type C. difficile 630*Δerm* and the isogenic *sigB* mutant strains, E. coli strain CA434 was used as a donor strain as previously described (48). C. difficile strains were cultured anaerobically at 37°C in either a Don Whitley VA-1000 or A55 workstation. Cells were cultured in brain heart infusion (BHI; Oxoid) broth supplemented with 0.5% (wt/vol) yeast-extract (BHIY) and 20 μg/ml thiamphenicol when appropriate. Unless additional antimicrobials/stressors were added (metronidazole Etest and sterile pads supplemented with different stressors), medium was supplemented with C. difficile selective supplement (CDSS; Oxoid).

The *sigB* ClosTron mutant described in this study was generated as described previously (49), using pMTL007C-E2_sigB171s::intron_ermB, synthesized by DNA2.0 (now ATUM). Design of the retargeted intron was performed with the Perutka algorithm, via the ClosTron website (http://clostron.com/). The mutant was verified using primers Cdi-sigB-F, Cdi-sigB-R, EBSuniversal, ErmRAM-F, and ErmRAM-R.

### Overproduction, purification and affinity purification of σ^B^_6×His_ for synthesis of a polyclonal anti-σ^B^ antibody.

**(i) Overproduction and purification of σ^B^_6×His_.** Overexpression of σ^B^_6×His_ was performed by using Escherichia coli Rosetta (DE3) pLysS cells (Novagen) harboring the E. coli expression plasmid pIB14. These cells were cultured in Luria-Bertani (LB) broth and induced with 0.5 mM isopropyl-β-d-thiogalactopyranoside (IPTG) for 1 h starting at an optical density of ≈0.6. Cells were collected by centrifugation at 4°C, and the resulting cell pellets were resuspended in lysis buffer (pH = 8.0; 50 mM NaH_2_PO_4_, 300 mM NaCl, 5 mM β-mercaptoethanol, 0.1% NP-40, and complete protease inhibitor cocktail [CPIC; Roche Applied Science]). Through the addition of 1 mg/ml lysozyme and sonication (6 × 20 s), cells were lysed. The lysate was drawn through a blunt 1.2-mm needle and was clarified by centrifugation at 13,000 × *g* at 4°C for 25 min. Recombinant σ^B^_6×His_ was purified from the supernatant on Talon Superflow resin (GE Healthcare) per the manufacturer’s instructions. Proteins were dialyzed and stored in buffer (pH = 8.0) containing 50 mM NaH_2_PO_4_, 300 mM NaCl, and 12% glycerol. Protein concentrations were determined using a Bradford assay (Bio-Rad). Two ml of σ^B^_6×His_ protein solution containing 2 mg/ml protein was sent to BioGenes GmbH (Berlin) for generation of a polyclonal rabbit anti-σ^B^ antibody.

**(ii) Affinity purification of the polyclonal anti-σ^B^ antibody.** Affinity purification of the antibody was performed to increase specificity of σ^B^ detection. Approximately 350 μg of purified σ^B^_6×His_ protein was loaded onto an SDS-PAGE gel. After electrophoresis and transfer of proteins to a polyvinylidene difluoride (PVDF) membrane using standard blotting procedures, purified σ^B^_6×His_ protein was visualized by Ponceau S staining, and the membrane containing the protein was cut as small as possible while retaining the region with the protein. The membrane was destained and washed with Tris-buffered saline with Tween 20 (TBST) buffer (500 mM NaCl, 20 mM Tris base, and 0.05% vol/vol Tween 20 [pH = 7.4]) twice for 5 min at room temperature. The membrane was then preeluted by soaking in acidic glycine solution (100 mM, pH = 2.5) for 5 min prior to washing with TBST twice for 5 min at room temperature. Subsequently, the membrane was blocked in 5% nonfat milk powder solution (Campina Elk, dissolved in TBST buffer) for 1 h at room temperature after again washing twice with TBST for 5 min. Serum containing anti-σ^B^ antibody was incubated on the membrane overnight at 4°C. After three 5-min washes with TBST, the membrane was washed twice for 5 min in PBS. Affinity-purified antibody was eluted from the membrane by adding acidic glycine solution and incubating for 10 min at room temperature. The pH of the eluate was adjusted to 7.0 through the addition of 1 M Tris-HCl (pH = 8.0). This step was repeated twice more, and the eluates were pooled and centrifuged (1 min at maximum speed) to remove precipitated protein and membrane particles. Bovine serum albumin (BSA) and sodium azide were added to the affinity-purified anti-σ^B^ antibody to end concentrations of 1 mg/ml and 5 mM, respectively, and the affinity-purified antibody was stored at −80°C.

### Characterization of the σ^B^ regulon.

**(i) σ^B^ overproduction in C. difficile.** Exponentially growing starter cultures of C. difficile strain IB58 and IB61 were diluted to an OD_600_ of 0.05 in BHIY medium supplemented with 20 μg/ml lincomycin and thiamphenicol (20 μg/ml) where appropriate. Cells were grown until an OD_600_ of ≈0.3, after which a 1-ml sample was taken for control by Western blotting, and gene expression was induced with 100 ng/ml ATc for 1 h. Subsequently, 1 ml of sample was taken for control by Western blotting, and 50 ml was collected by centrifugation and stored at −20°C until RNA extraction. Noninduced samples were treated and collected identically, except that no ATc was added at an OD_600_ ≈0.3. All samples were corrected for OD_600_ prior to analysis by Western blot.

**(ii) RNA extraction.** Bacterial RNA was extracted and analyzed as previously described (50). Briefly, cell pellets were lysed for 30 min at room temperature in enzymatic lysis buffer consisting of 15 mg/ml lysozyme and Tris-EDTA (TE) buffer. Further disruption of cells was performed by vigorous mechanical lysis for 3 min in RLT buffer to which one spatula of glass beads was added. After samples were centrifuged (3 min at 10,000 rpm at 4°C) and 100% ethanol was added to the supernatant, RNA was purified using the Qiagen RNeasy kit protocol according to manufacturer’s instructions. DNA contamination was removed by using RNase-free DNase I (Qiagen) twice prior to elution of the RNA samples in H_2_O. RNA quality and integrity numbers (RINs) were assessed with a Bioanalyzer 2100 (Agilent) and RNA 6000 Nano reagents (Agilent). Only samples with an RIN of ≥7 were used for further analysis.

**(iii) DNA microarray and data analysis.** A customized whole-genome DNA microarray of the 630Δ*erm* strain was used (8 × 15K format; Agilent) (50). Quadruplicate samples were analyzed for the DNA microarray. Using the ULS fluorescent labeling kit for Agilent arrays (Kreatech), 1 μg of total RNA was used for labeling with either Cy3 (P_tet_-*sigB*) or Cy5 (P_tet_-*sluc^opt^*). After pooling and fragmentation, 300 ng of labeled RNA per sample was hybridized according to the two-color microarray protocol from Agilent. DNA microarrays were scanned with an Agilent C scanner and analyzed as described previously (50). A gene was considered differentially expressed if the log_2_ fold change (log_2_ FC) was ≤−1.5 or ≥1.5 and the *P* value was <0.05. Results were visualized in VolcaNoseR (28) and are available as an interactive graph via the URL contained in Text S1 in the supplemental material.

### *In vitro* transcription.

DNA oligonucleotides oWKS-1506 and oWKS-15136 (64 and 82 bp, respectively) were end labeled with ɣ-32^P^-ATP using T4 polynucleotide kinase (PNK; Invitrogen) and used as a size indicator for the *in vitro* transcription reactions. For the end labeling reaction, 1 μl ɣ-^32^P-ATP was incubated together with 200 pmol oligonucleotide and 1 μl (10 U) PNK in Forward reaction buffer (70 mM Tris-HCl [pH 7.6], 10 mM MgCl_2_, 100 mM KCl, and 1 mM 2-mercaptoethanol) at 37°C for 30 min. For the *in vitro* runoff transcriptions, sigma factors and RNA polymerase core enzyme were preincubated with PCR-amplified promoter areas (for P*_CD0350_*, P*_CD0872_*, P*_CD2963_*, P*_CD3412_*, P*_CD3605_*, and P*_CD3614_*) or XbaI-linearized pCD22 (P*_tcdA_*) for 30 min at 37°C prior to the start of the reaction. PCR products of the promoter areas as used for the *in vitro* transcription reactions were loaded on and excised from agarose gels and purified using a NucleoSpin gel and PCR clean-up kit (Macherey-Nagel). *In vitro* transcription reactions mixtures contained 1 μl (1 U) E. coli RNAP^core^ (catalog no. M0550S; NEB), 16 pmol sigma factor, 0.5 pmol DNA, 10 mM nucleoside triphosphate (NTP) mix, and 0.3 μl α-^32^P-ATP in reaction buffer (40 mM Tris-HCl, 150 mM KCl, 10 mM MgCl_2_, 1 mM dithiothreitol [DTT], and 0.01% Triton X-100 [pH = 7.5]) and were incubated for 15 min at 37°C. Transcripts and labeled oligonucleotides to be used as a size indication were purified using P-30 Bio-Gel spin columns (Bio-Rad). All reactions were stopped in gel loading buffer II (Invitrogen) containing 95% formamide, 18 mM EDTA, and 0.025% each of SDS, xylene cyanol, and bromophenol blue at 95°C for 5 min and loaded on 8% monomeric UreaGel (SequaGel; National Diagnostics). Gels were dried and exposed to phosphorimager screens overnight (approximately 17 h) and imaged with a Typhoon 9410 scanner (GE Healthcare).

### Spot assay for viability upon σ^B^ overproduction in C. difficile and vector stability assay.

C. difficile overnight precultures were corrected for OD_600_ and were subsequently 10-fold serially diluted in BHI medium. Spots (2 μl) of each dilution were plated on selective (20 μg/ml thiamphenicol) and unselective square (90 × 90 × 15 mm; VWR international) BHI plates with or without 200 ng/ml anhydrotetracycline (ATc). Growth was evaluated after 24 h, and swabs were subsequently taken from all strains grown on unselective BHI agar plates with and without 200 ng/ml ATc for the vector stability assay. These swabs used for the vector stability assay were resuspended in PBS to a McFarland turbidity of 1.0, adjusted for their OD_600_ values, and 10-fold serially diluted in nonselective BHI medium. Of these serially diluted suspensions, 10-μl spots of each dilution were then plated on selective (20 μg/ml thiamphenicol plus CDSS) and nonselective (BHI plus CDSS) plates, and CFU/ml was counted after 24 to 48 h of growth. The percentage of cells retaining the plasmid was calculated as (CFU/ml)_selective_/(CFU/ml)_nonselective_ × 100%. If no growth was detected on selective plates containing thiamphenicol, the percentage of plasmid maintained was set as 0%. To calculate statistical significance between percent plasmid maintained in strains induced or not induced by ATc, an unpaired Student’s *t* test was used.

### Plate-based luciferase assay with metronidazole Etest and disk diffusion.

Strains harboring luciferase reporter plasmids were grown on prereduced, selective BHI plates for 24 h. Subsequently, bacterial suspensions corresponding to 1.0 McFarland turbidity were applied on BHI agar supplemented with 0.5% yeast extract, after which a metronidazole Etest or plain disks were applied. Disks were spotted with 10 μl each of sterile H_2_O, 1 M H_2_O_2_, 3,000 μg/ml lincomycin, 200 μg/ml metronidazole, 400 μg/ml ibezapolstat, and 200 μg/ml vancomycin. After 24 h of growth, luciferase activity was visualized by spraying 1:100 reconstituted NanoGlo luciferase substrate (catalog no. N1110; Promega) on the agar plate using a disposable spray flask. One spray corresponded to approximately 250 μl reconstituted NanoGlo luciferase substrate. Luminescence was recorded using a Uvitec Alliance Q9 Advanced imager (BioSPX) after a 10-s exposure time per plate. Luciferase was conjugated into a *sigB* knockout made by allelic coupled exchange (51), whereas a ClosTron mutant background was used for the DNA arrays. However, no differences between these backgrounds have ever been observed in our assays.

### Data availability.

The data used in the VolcaNoseR visualization have been deposited at Zenodo for this purpose (https://doi.org/10.5281/zenodo.3945936). Full transcriptome data have been deposited in the GEO database and can be accessed through the identifier GSE152515.
