# Supplementary material for: Redefining the Clostridioides difficile σB Regulon: σB Activates Genes Involved in Detoxifying Radicals That Can Result from the Exposure to Antimicrobials and Hydrogen Peroxide
Source: mSphere. 2020 Sep 16;5(5):e00728-20. doi: 10.1128/mSphere.00728-20 (PMC7494833; doi:10.1128/mSphere.00728-20)
Supplement: TEXT S1 [file mSphere.00728-20-s0001.pdf]

LINK:

https://huygens.science.uva.nl:/VolcaNoseR/?data=5;;log2FC;significance;locus_tag&vis=4;0.5;-1.5,1.5;1.3;significant;manh&can=10;;;CD630DERM_02390,CD630DERM_02430,CD630DERM_02290,CD630DERM_02500&layout=;;TRUE;-5,10;0,8;X;600;800&color=1;none&label=TRUE;Differential expression (sigB OE/ctrl) in the presence of thiamphenicol;TRUE;log2(FC);-10log(adj_Pvalue);TRUE;24;24;18;4;&url=https://tinyurl.com/y9abwbsb

BACKUP LINK:

https://huygens.science.uva.nl:/VolcaNoseR2/?data=5;;log2FC;significance;locus_tag&vis=4;0.5;-1.5,1.5;1.3;significant;manh&can=10;;;CD630DERM_02390,CD630DERM_02430,CD630DERM_02290,CD630DERM_02500&layout=;;TRUE;-5,10;0,8;X;600;800&color=1;none&label=TRUE;Differential expression (sigB OE/ctrl) in the presence of thiamphenicol;TRUE;log2(FC);-10log(adj_Pvalue);TRUE;24;24;18;4;&url=https://tinyurl.com/y9abwbsb

ALTERNATIVE BACKUP LINK:

http://goedhart.shinyapps.io/VolcaNoseR/?data=5;;log2FC;significance;locus_tag&vis=4;0.5;-1.5,1.5;1.3;significant;manh&can=10;;;CD630DERM_02390,CD630DERM_02430,CD630DERM_02290,CD630DERM_02500&layout=;;TRUE;-5,10;0,8;X;600;800&color=1;none&label=TRUE;Differential expression (sigB OE/ctrl) in the presence of thiamphenicol;TRUE;log2(FC);-10log(adj_Pvalue);TRUE;24;24;18;4;&url=https://tinyurl.com/y9abwbsb
